# Genome editing of *Ralstonia eutropha* using an electroporation-based CRISPR-Cas9 technique

**DOI:** 10.1186/s13068-018-1170-4

**Published:** 2018-06-20

**Authors:** Bin Xiong, Zhongkang Li, Li Liu, Dongdong Zhao, Xueli Zhang, Changhao Bi

**Affiliations:** 1 University of Chinese Academy of Sciences, Beijing, 100049 People’s Republic of China; 2 Tianjin Institute of Industrial Biotechnology, Chinese Academy of Sciences, Tianjin, 300308 People’s Republic of China; 3 Key Laboratory of Systems Microbial Biotechnology, Chinese Academy of Sciences, Tianjin, 300308 People’s Republic of China; 4 University of Sciences and Technology of China, Hefei, 230026 People’s Republic of China

**Keywords:** *Ralstonia eutropha*, *Cupriavidus necator*, Electroporation, CRISPR, Cas9, Genome editing

## Abstract

**Background:**

*Ralstonia eutropha* is an important bacterium for the study of polyhydroxyalkanoates (PHAs) synthesis and CO_2_ fixation, which makes it a potential strain for industrial PHA production and attractive host for CO_2_ conversion. Although the bacterium is not recalcitrant to genetic manipulation, current methods for genome editing based on group II introns or single crossover integration of a suicide plasmid are inefficient and time-consuming, which limits the genetic engineering of this organism. Thus, developing an efficient and convenient method for *R. eutropha* genome editing is imperative.

**Results:**

An efficient genome editing method for *R. eutropha* was developed using an electroporation-based CRISPR-Cas9 technique. In our study, the electroporation efficiency of *R. eutropha* was found to be limited by its restriction-modification (RM) systems. By searching the putative RM systems in *R. eutropha* H16 using REBASE database and comparing with that in *E. coli* MG1655, five putative restriction endonuclease genes which are related to the RM systems in *R. eutropha* were predicated and disrupted. It was found that deletion of *H16_A0006* and *H16_A0008*-*9* increased the electroporation efficiency 1658 and 4 times, respectively. Fructose was found to reduce the leaky expression of the arabinose-inducible pBAD promoter, which was used to optimize the expression of *cas9*, enabling genome editing via homologous recombination based on CRISPR-Cas9 in *R. eutropha*. A total of five genes were edited with efficiencies ranging from 78.3 to 100%. The CRISPR-Cpf1 system and the non-homologous end joining mechanism were also investigated, but failed to yield edited strains.

**Conclusions:**

We present the first genome editing method for *R. eutropha* using an electroporation-based CRISPR-Cas9 approach, which significantly increased the efficiency and decreased time to manipulate this facultative chemolithoautotrophic microbe. The novel technique will facilitate more advanced researches and applications of *R. eutropha* for PHA production and CO_2_ conversion.

**Electronic supplementary material:**

The online version of this article (10.1186/s13068-018-1170-4) contains supplementary material, which is available to authorized users.

## Background

*Ralstonia eutropha* H16, also known as *Cupriavidus necator* H16, is a Gram-negative β-proteobacterium that is ubiquitously present in soil and freshwater environments [1]. It has attracted considerable research interest due to its significant economic potential [2] and CO_2_ fixation ability [3, 4]. This facultative chemolithoautotrophic bacterium is a metabolically versatile organism that can grow well under both lithoautotrophic and heterotrophic conditions [1]. Under lithoautotrophic conditions, it fixes CO_2_ via the Calvin–Benson–Bassham (CBB) cycle, which comprises enzymes encoded by the two CBB operons [1]. The energy used to implement CO_2_ fixation and maintain cell growth is generated by energy-conserving hydrogenases, which oxidize molecular H_2_ and thereby reduce NAD^+^ to form NADH [1]. The lithoautotrophic *R. eutropha* has great potential as a chassis for the study of CO_2_ fixation and development of microbial cell factories for syngas utilization. Under heterotrophic conditions, several organic substrates, such as fructose, gluconate, N-acetylglucosamine and some organic acids, are utilized as carbon sources [1]. *R. eutropha* grows to very high cell densities (281 g/L) under nutritionally rich conditions, and accumulates large amounts of PHAs (232 g/L) when the nitrogen or phosphate source is limited [2]. This characteristic makes it an attractive host for the synthesis of industrially relevant PHA materials.

In recent years, *R. eutropha* was engineered to produce biofuels, such as branched-chain alcohols [3], methyl ketones [5], hydrocarbons [6] and isopropanol [7]. In addition, *R. eutropha* can also be cultured under lithoautotrophic conditions to produce PHAs [8, 9] and biofuels [3, 5]. Although *R. eutropha* can already be engineered, convenient and efficient synthetic-biology tools are still underdeveloped compared to those available for model organisms such as *Escherichia coli* and *Saccharomyces cerevisiae*, and current manipulation techniques are still inefficient and time-consuming [10]. One of the major reasons for this is the low transformation efficiency of *R. eutropha* [11]. While common heat-shock transformation is not feasible in *R. eutropha,* electroporation has also been used rarely in previous publications, due to its extraordinarily low efficiency, which is several orders of magnitude lower than that of *E. coli* [11]. These properties make cell-to-cell transconjugation almost the only way to transfer plasmids into *R. eutropha,* which is not ideal for genome manipulation.

There are currently two techniques for genome editing of *R. eutropha*, one of which is based on group II introns, which is complicated and is consequently rarely used [10]. Another method was designed to integrate a suicide plasmid, via a single crossover recombination event [12, 13]. The integrating plasmid is transferred to *R. eutropha* via conjugation from a special host—*E. coli* S17-1 [14]. Transconjugants carrying the integrated plasmid are selected using proper antibiotics, and strains that have lost the integration vector via a second single crossover are selected in rich medium containing sucrose using *sacB* as the negative selection marker. This method which would take an average of 2–3 weeks to delete a single gene is not only time-consuming, but is also not very efficient [13].

Microbial genome editing techniques have progressed significantly due to the extensive research conducted on the CRISPR system (clustered regularly interspaced short palindromic repeats), derived from the RNA-guided immune systems found in many bacteria and archaea [15–17]. CRISPR-Cas9 and CRISPR-Cpf1 are Class 2 CRISPR-Cas systems, and are further classified as types II and V, respectively [18]. The systems recognize unique sequences and generate double strand breaks (DSBs) at the target locus, after which the DSBs is repaired either through NHEJ or HR [17]. CRISPR-Cas assisted genome editing tools have already been developed for a number of bacteria, including but not limited to *Streptococcus pneumoniae* [16], *E. coli* [19], *Streptomyces* [20, 21], *Lactobacillus reuteri* [22], *Clostridium* [23], *Bacillus subtilis* [24] and *Corynebacterium glutamicum* [25, 26]. However, this technique has not been developed in *R. eutropha* to date. Thus, we aimed to develop a convenient and efficient CRISPR-Cas assisted genome editing method for *R. eutropha*, preferentially using fast transformation methods, omitting the need for conjugation.

## Methods

### Strains and culture conditions

*Escherichia coli* S17-1 [14] was used for plasmid maintenance and conjugation with *R. eutropha*, and was cultured at 37 °C in Luria–Bertani medium (LB, 10 g/L tryptone, 5 g/L yeast extract, 10 g/L NaCl) with 100 µg/mL streptomycin or 50 µg/mL kanamycin if necessary. *R. eutropha* H16 [1] was the parent strain for genetic modifications and was cultured aerobically at 30 °C in LB with 10 µg/mL gentamicin or 200 µg/mL kanamycin for plasmid maintenance. All strains used in this study are listed in Table 1.

**Table 1:** List of strains used in this study

Strain	Description	Source or references
*E. coli*		
S17-1	Host strain for transconjugation, *thi pro recA hsdR* [RP4-2Tc::Mu-Km::Tn7] Tp^r^ Sm^r^	Laboratory stock
MG1655	Wild type	Laboratory stock
MG18	Derived from MG1655, *poxB*::*H16_A0004*-*5*	This study
*R. eutropha*		
H16	Wild type, Gen^r^	ATCC 17669
C1	H16∆*H16_A0006*	This study
C2	H16∆*H16_A0008*-*9*	This study
C3	H16∆*H16_A0014*	This study
C4	H16∆*PHG170*	This study
C5	H16∆*H16_A0006*∆*H16_A0008*-*9*	This study
C5rfp	C5, *phaP1*::*rfp*	This study

### Plasmid construction

Primers (Additional file 1: Table S4) were designed using the j5 DeviceEditor [27] and synthesized by Genewiz (Beijing, China). DNA polymerase, BsaI restriction endonuclease and T4 ligase were purchased from Takara (Dalian, China), New England Biolabs (USA) and Thermo-Fisher Scientific (USA), respectively. The plasmids used in this study were constructed via Golden Gate [28] or Gibson [29] assembly, and cloned directly into *E. coli* S17-1. Plasmids used in this study are in Additional file 1: Table S3.

### Plasmid extraction

Plasmids in *E. coli* or *R. eutropha* were extracted using AxyPrep Plasmid Miniprep Kit (AXYGEN, China) according to the manuscript with some minor modifications. For *R. eutropha*, after extraction reagents were added to the collected sample from 1 mL LB medium and the mixture was centrifuged at 13,000×*g* for 20 min. While for *E. coli,* centrifugation was performed at 12,000×*g* for 10 min. To facilitate the following step of electroporation, the eluent solution of the kit was replaced by sterile deionized water for plasmid elution. The plasmid solution obtained from the adsorbing column was used for eluting another column to improve the plasmid concentration.

### Preparation of competent cells

For *E. coli*, the procedure was performed using a previously described protocol [19]. To prepare *R. eutropha* competent cells, the method described by Hae-Chul Park [11] was employed with some modifications. The procedure used in this study was performed as follows. An aliquot of a glycerol cryopreservation stock was streaked onto an LB plate with 10 µg/mL gentamicin and incubated for 48 h at 30 °C, after which a single colony was picked up and incubated aerobically in LB medium at 30 °C and 200 rpm. The resulting seed culture was transferred into 100 mL LB and cultured to an optical density at 600 nm (OD_600_) of 0.6–0.8, after which it was chilled on ice for 5–10 min. Cells were harvested by centrifugation at 3000×*g* and 4 °C for 5 min and washed three times with ice-cold sterile 10% glycerol. The cell pellet collected from 100 mL bacteria solution was resuspended in 0.6 mL 10% glycerol and aliquoted into sterile 1.5 mL tubes. Then the competent cells were used immediately or frozen in liquid nitrogen and preserved at − 80 °C.

### Conjugation and electroporation

Conjugation was performed as follows. The *E. coli* S17-1 donor harboring the transferable plasmid was cultured in 10 mL LB at 37 °C for 12 h, and the *R. eutropha* recipient was cultured in 10 mL LB at 30 °C for 24 h, before they were mixed and centrifuged at 3000×*g* for 5 min. The supernatant was removed and the cell pellet was washed with 30 mL LB, after which the cells were resuspended in 100 µL LB, dropped onto LB plates without antibiotics and incubated at 30 °C for 24 h. Subsequently, a portion of the mixed bacterial lawn was resuspended in LB, plated on LB agar plates with 200 µg/mL kanamycin and 10 µg/mL gentamicin, and incubated at 30 °C for 48 h.

For the electroporation of *R. eutropha*, 8 µL of high-quality plasmid DNA (~ 400 ng) was added to 100 µL of competent cells, and transferred into a pre-chilled 2-mm electroporation cuvette (Bio-Rad, USA), and incubated on ice for 5 min, after which electroporation was performed at a voltage of 2.3 kV. Immediately afterward, 1 mL LB with 10 mg/mL fructose was added to the cells, and the resulting suspension was transferred to a sterile 1.5 mL centrifuge tube. After incubation at 30 °C for 2 h, the cells were spread on LB agar plates with 200 µg/mL kanamycin and 10 mg/mL fructose, and incubated at 30 °C for 48 h.

### Gene knockout (or integration) via pK18mobsacB

Two homologous templates which were ~ 500 bp, respectively, were cloned into the pK18mobsacB plasmid backbone via Golden Gate [28] or Gibson [29]. For integration, the target gene was cloned between the two homologous templates. The knockout plasmid was transformed into *E. coli* S17-1, then identified and transferred to *R. eutropha* via conjugation. Single colonies were cultured in LB with 10 µg/mL gentamicin and 200 µg/mL kanamycin at 30 °C. A pair of primers with one bound to the genome and another to the plasmid was used for colony PCR to identify strains with the knockout plasmid integrated. Then corrected strains were incubated in LB without NaCl and kanamycin, but with 100 mg/mL sucrose at 30 °C for 72 h. Strains were streaked on LB plate (without NaCl and kanamycin, but with 50 mg/mL sucrose), then resistance against kanamycin was investigated, and single colonies with no resistance against kanamycin were identified by PCR. *H16_A0006*, *H16_A0008*-*9*, *H16_A0014*, *PHG170* deletion and *rfp* integration were performed by this method.

### Plasmid for gene editing via CRISPR-Cas system

All plasmids used for *R. eutropha* gene editing via CRISPR-Cas system were derived from the broad-range-host plasmid pBBR1MCS2 [30] and constructed via Golden Gate. The *csa9* gene was amplified from the plasmid pCas9 (Addgene number 42876) [16, 19] and driven by the arabinose-inducible pBAD promoter, while the corresponding sgRNA was transcribed from a constitutive promoter (ctaggtttatacataggcgagtactctgttatggagtcagatcttagc). 20 bp sequence (take *rfp* for example, catgcgtttcaaagttcgta) was selected as the guide sequence. The *cpf1* gene was amplified from the plasmid pFnCpf1_min (Addgene number 69975) [31] and driven by the arabinose-inducible pBAD promoter, while the corresponding sgRNA was transcribed from the constitutive promoter BBa_J23109. A 24 bp sequence (take *rfp* for example, caaagttcgtatggaaggttccgt) was selected as the guide sequence. Genes *ligD* and *ku70* were amplified from pCas9(Ts)-NHEJ [32] provided by Prof. Qingsheng Qi (Shandong University, Ji’nan).

### Genome editing via CRISPR-Cas9

Plasmid for gene editing was amplified in *E. coli* S17-1 and electroporated into *R. eutropha*. A single colony from the transformation plate was cultured in LB with 2 mg/mL arabinose and 200 µg/mL kanamycin for 120–168 h to induce the editing process, after which the cells were spread on LB plates with the same concentrations of arabinose and kanamycin to identify edited strains by colony PCR. Afterward, plasmid curing was performed by growing the cells in LB without kanamycin at 30 °C for 24 h and confirmed by testing for loss of resistance against kanamycin.

## Results and discussions

### Enhancing electroporation efficiency of *R. eutropha* by identification and deletion of key restriction endonuclease genes

**Fig. 1:**
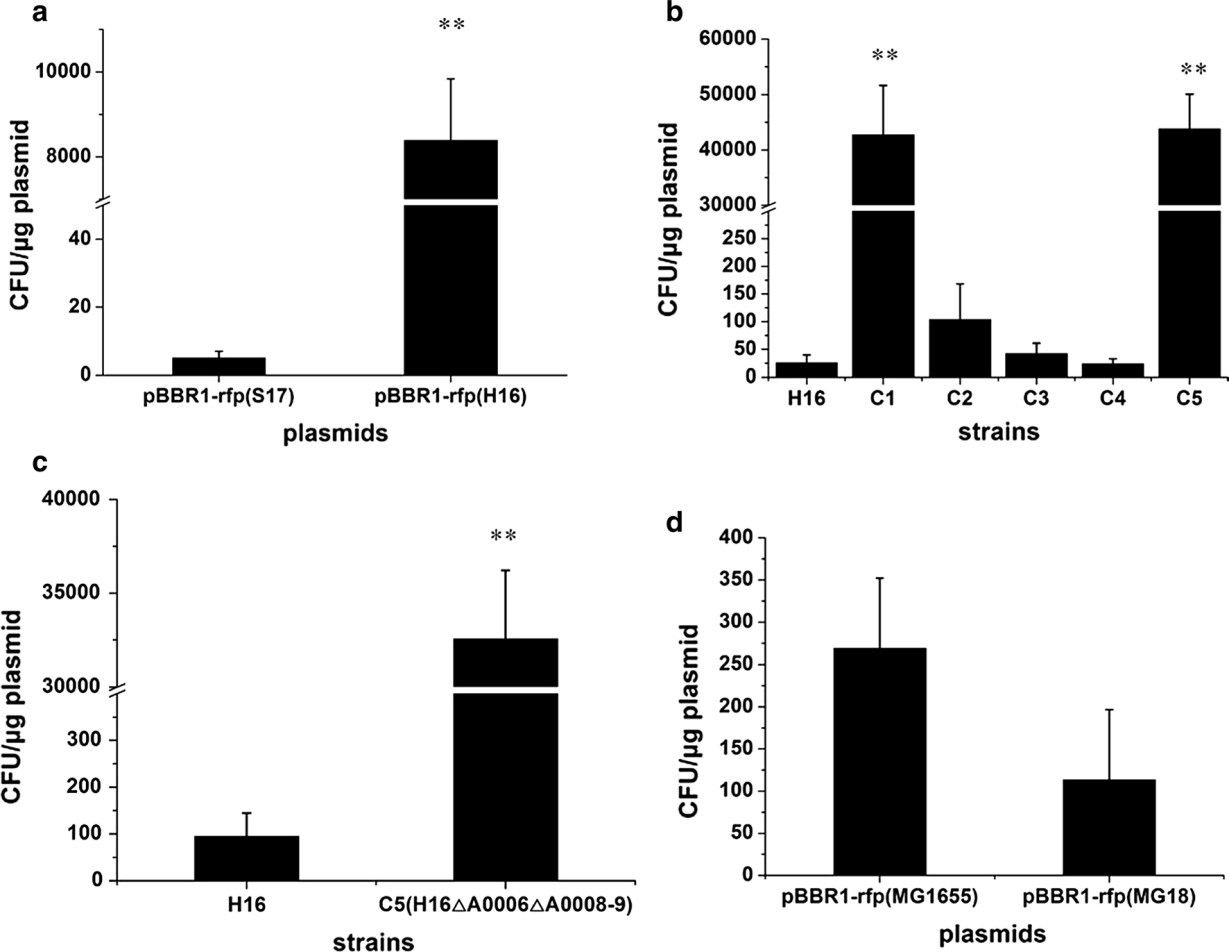
Electroporation efficiencies of different plasmid DNA in various *R. eutropha* strains. **a** Electroporation efficiencies of pBBR1-rfp extracted from *E. coli* S17-1 or *R. eutropha* H16 in *R. eutropha* H16. pBBR1-rfp(S17), pBBR1-rfp extracted from *E. coli* S17-1. pBBR1-rfp(H16), pBBR1-rfp extracted from *R. eutropha* H16. **b** Electroporation efficiencies of pBBR1-rfp in H16 and engineered *R. eutropha* strains. pBBR1-rfp was extracted from *E. coli* S17-1. **c** Electroporation efficiencies of pBBR1-rfp(NM) in *R. eutropha* H16 and C5. pBBR1-rfp(NM), non-methylated pBBR1-rfp. **d** Electroporation efficiencies of pBBR1-rfp from *E. coli* MG1655 and MG18 in *R. eutropha* H16. pBBR1-rfp(MG1655), pBBR1-rfp extracted from *E. coli* MG1655. pBBR1-rfp(MG18), pBBR1-rfp extracted from *E. coli* MG18. All experiments were repeated four times and the error bars represent standard deviations. The significance of differences was calculated by one-way ANOVA using SPSS18.0 software. Asterisks indicate a significant difference compared with the control (**p < 0.01, highly significant difference, *p < 0.05, significant difference)

One of the major problems hindering the genome editing of *R. eutropha* is its low electroporation efficiency. To identify the main reasons for this problem, the plasmid pBBR1-rfp was transferred into either *E. coli* S17-1 or *R. eutropha* H16, extracted and electroporated into *R. eutropha* H16 again. This plasmid was derived from the broad-host-range plasmid pBBR1MCS2 [30], by introducing a red fluorescent protein (*rfp*) gene driven by the constitutive promoter BBa_J23100, which made its carriers exhibit visible red color. Intriguingly, it was found that the electroporation efficiency of pBBR1-rfp extracted from *R. eutropha* was 1677 times higher than that of its counterpart from *E. coli* S17-1 (Fig. 1a). The two plasmids had the same DNA sequence, but were likely modified by different RM systems in *E. coli* and *R. eutropha*, which indicated that the *R. eutropha* RM systems may be the major cause of the low electroporation efficiency.

To investigate this hypothesis, we searched the genome of *R. eutropha* using the prediction tool based on the REBASE database [33] and identified four bona fide RM systems. Within these five putative restriction endonuclease genes, *H16_A0006*, *H16_A 0008*, *H16_A0009*, *H16_A0014* and *PHG170* (*H16_A0008* and *H16_A0009* may encode two subunits of a single endonuclease) were predicted by comparing with the RM systems of *E. coli* MG1655 (Additional file 1: Table S1). To determine their effects on the transformation efficiency, the putative restriction endonuclease genes were knocked out individually, yielding four *R. eutropha* RM knockout strains C1, C2, C3 and C4. The electroporation efficiencies of the wild-type strain H16 and the four knockout strains were tested using the plasmid pBBR1-rfp extracted from *E. coli* S17-1. The results showed that while C3 and C4 did not show an obvious enhancement of electroporation efficiency, the efficiencies of the strains C2 and C1 were, respectively, improved 4 and an astonishing 1658 times over H16 (Fig. 1b). This result indicated that endonucleases encoded by the genes *H16_A0006* and *H16_A0008*-*9* were indeed the major reason for the low transformation efficiency of *R. eutropha*. Subsequently, strain C5 was constructed by disrupting the *H16_A0008*-*9* gene in strain C1, which led to an electroporation efficiency that was 1697 times higher than that of H16 strain, even if no significant improvement was evident compared to C1 (Fig. 1b). *mcrBC* in *E. coli*, which is homologous to the *H16_A0008*-*9* in *R. eutropha* H16, was disrupted to enhance transformation efficiency in some laboratory strains [34], such as DH10B, DH12S, DM1, and HB101. Therefore, the C5 strain with a similar double knockout was selected for future research.

To investigate the electroporation efficiency of an in vitro constructed plasmid with no methylation, linear pBBR1-rfp was generated by PCR and ligated using the Golden Gate assembly method to obtain non-methylated plasmid DNA of pBBR1-rfp(NM). The resulting non-methylated material was individually electroporated into C5 and H16, which revealed that the electroporation efficiency of C5 with in vitro constructed DNA was 343 times higher than that of H16 (Fig. 1c). Thus, the deletion of *H16_A0006* or *H16_A0006* along with *H16_A0008*-*9* enabled the efficient electrotransformation of *R. eutropha*, regardless of the methylation status of the plasmid DNA.

On the other hand, the genes *H16_A0004*-*5*, which are adjacent to *H16_A0006*, were predicted to encode the putative methyltransferase and specificity subunits of the RM system. Hence, the MG18 strain was constructed by integrating *H16_A0004*-*5* with the constitutive promoter BBa_J23100 and an RBS into the *poxB* loci of the *E. coli* MG1655 genome, which should have enabled it to methylate plasmids according to the methylation-protection pattern of *R. eutropha*. However, when pBBR1-rfp extracted from MG1655 and MG18 were electroporated into *R. eutropha* H16, there was no significant difference in electroporation efficiency (Fig. 1d). These results indicated that *H16_A0004*-*5* expressed in *E. coli* MG1655 alone is not sufficient to properly methylate the plasmid DNA in this study.

### Optimal expression strategy with fructose as the suppression factor

**Fig. 2:**
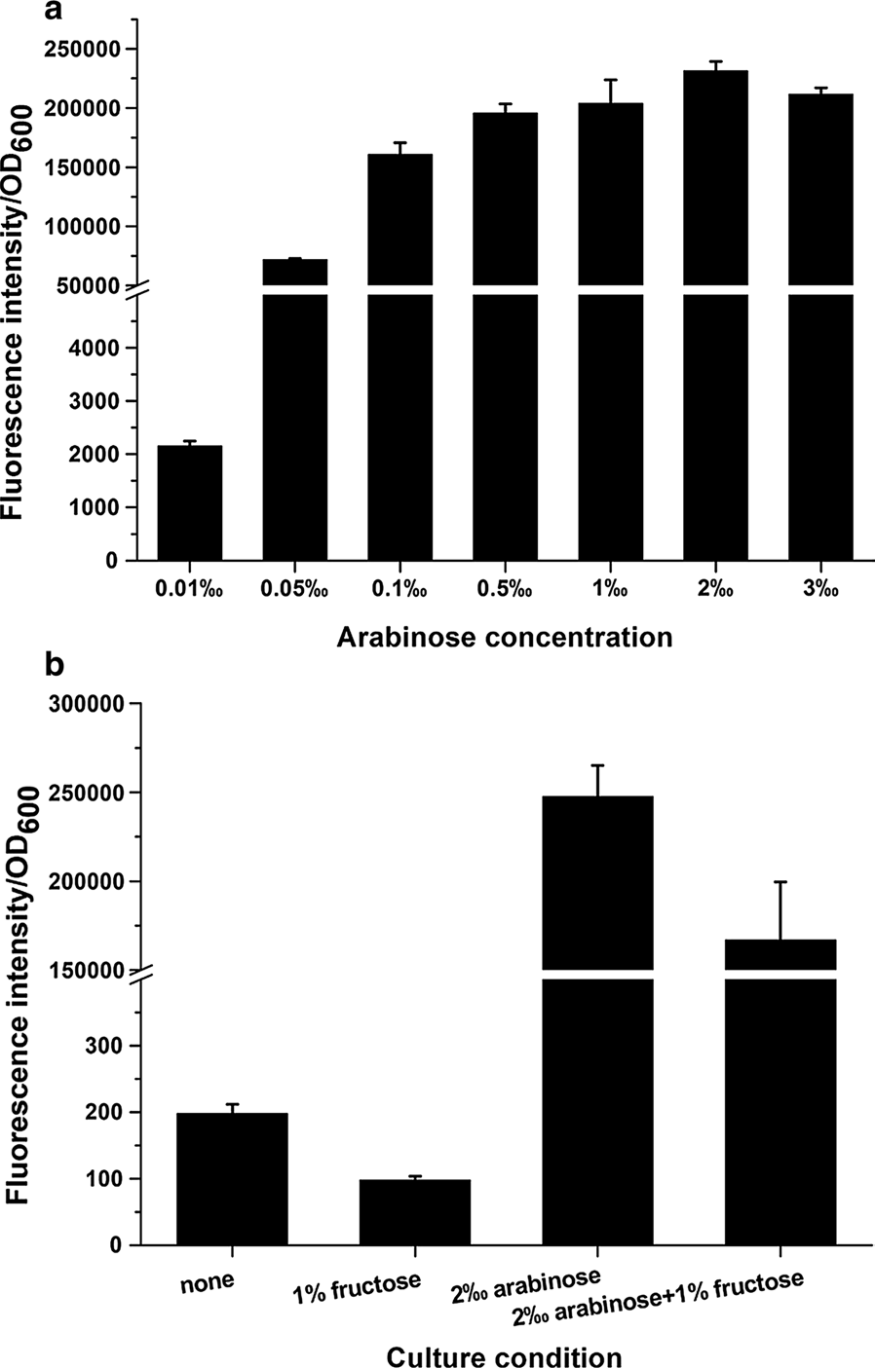
Expression levels of pBAD-controlled *rfp* under different induction conditions. **a** Expression levels of pBAD-*rfp* induced by different concentrations of l-arabinose. **b** Expression levels of pBAD-*rfp* suppressed by fructose. All experiments were performed in LB and repeated three times to obtain mean values and standard deviations

Due to the relatively low transformation efficiency of *R. eutropha*, even in case of the C5 strain, linear DNA could not be used as the donor template for genome editing. To simplify the transformation process, a single plasmid was employed to carry all the functional parts of the CRISPR-Cas-based editing tool. The precise expression control of *cas9* and sgRNA obtained with this strategy was the key for successful genome editing [19]. In this study, the arabinose-inducible pBAD promoter was selected to express the *cas* genes (*cas9* and *cpf1* [31]). pBBR1-pBAD-rfp [6] carrying pBAD-controlled *rfp* was used to transform the C5 strain to investigate the optimal expression level, which increased with the increase of l-arabinose concentration in LB, and the maximal expression was achieved at 2 mg/mL l-arabinose (Fig. 2a). Under these conditions, the *rfp* expression level was three orders of magnitude higher than in the control (Fig. 2b), which should be suitable for the expression of the *cas* genes, which are large (~ 4 kb) and have an extremely low GC content (~ 30%). In contrast to *E. coli*, glucose did not suppress the basal expression of pBAD, since it is not transported into *R. eutropha* H16 [35]. Interestingly, we found that the expression of pBAD was significantly suppressed by fructose (Fig. 2b). Hence, an expression strategy was developed that used pBAD as the inducible promoter for the expression of the *cas* genes, and fructose was added to decrease its basal expression.

### Development of the CRISPR-Cas9 genome editing method for *R. eutropha*

To develop an efficient genome editing method for *R. eutropha*, both CRISPR-Cas9 and CRISPR-Cpf1 systems were evaluated. The systems were individually cloned into the pBBR1MCS2 vector backbone using Golden Gate or Gibson assembly. The expression of *cas9* or *cpf1* on the plasmids was driven by the pBAD promoter, while the corresponding sgRNA was transcribed from a constitutive promoter. By searching the genome sequence, *H16_B2352* and *H16_B2355* were found to encode gene products with 40 and 28% similarity to LigD and Ku70 from *Mycobacterium tuberculosis* H37Rv (Additional file 1: Table S2), which may encode a putative ATP-dependent DNA ligase and non-homologous end-binding protein, respectively [36]. This indicated that *R. eutropha* may possess a native NHEJ mechanism. To investigate whether NHEJ-based editing mediated by the CRISPR-Cas system is functional in the strain, *rfp* was integrated into the downstream of *phaP1* to obtain the strain C5rfp, which exhibits red fluorescence and can be conveniently detected using a microplate reader, whereby the red fluorescence would disappear if the *rfp* gene was disrupted. The two plasmids pBBR1-Cas9 and pBBR1-Cpf1, respectively, harboring the CRISPR-Cas9 and CRISPR-Cpf1 systems, were constructed, confirmed and transferred into *R. eutropha* via electroporation or conjugation. It should be noted that the electroporation and conjugation efficiencies of pBBR1-Cas9 and pBBR1-Cpf1 were significantly lower than those of pBBR1-rfp, which were about two to three orders of magnitude lower than the latter. Moreover, few transformants were acquired by electroporation when fructose was not added, which may be due to the large size of the plasmids (> 9 kb) and leaky expression of the *cas* genes. However, when 10 mg/mL fructose was added to the regeneration medium in the process of electroporation, sufficient numbers of transformants were obtained, although the efficiency which was less than 10^2^ CFU/µg of plasmid DNA was still lower than that of pBBR1-rfp. Transformants identified by PCR and sequencing were cultured in LB with 2 mg/mL arabinose and 200 µg/mL kanamycin for 120–168 h, after which they were streaked onto LB plates with arabinose and kanamycin at the same concentrations. 48 single colonies were picked randomly and red fluorescence was detected with a microplate reader, while C5rfp and C5 were selected as positive and negative controls, respectively. The result indicated that none of the *rfp* genes was disrupted (Table 2), which therefore showed that CRISPR-Cas systems and the native NHEJ mechanism alone were not able to edit the target gene in *R. eutropha*.

**Table 2:** Editing efficiencies of *rfp* via different CRISPR-Cas systems and repair mechanisms

CRISPR-Cas system	DSB repair	Red colonies	Colorless colonies	Total^a^	Efficiency (%)
CRISPR-Cas9	Native NHEJ	48	0	48	0
	Heterologous NHEJ	48	0	48	0
	HR^b^	0	23	23	100
CRISPR-Cpf1	Native NHEJ	48	0	48	0
	Heterologous NHEJ	48	0	48	0
	HR	23	0	23	0

^a^Colonies were picked randomly and red fluorescence was measured on a microplate reader

^b^Colonies were picked randomly and identified by PCR. One of the PCR products was verified by sequencing

To introduce a heterologous NHEJ mechanism into *R. eutropha*, the *ligD* and *ku70* genes from *M. tuberculosis* H37Rv were cloned together with the constitutive promoter BBa_J23119 into both pBBR1-Cas9 and pBBR1-Cpf1, to construct the plasmids pBBR1-Cas9-ligD-ku and pBBR1-Cpf1-ligD-ku [32]. The two plasmids were individually transferred into C5rfp and the resulting strain was induced under the conditions described above. However, while 48 single colonies were obtained, none of them had disruptions of the *rfp* genes (Table 2), which indicated that no editing events occurred with the CRISPR-Cas and heterologous NHEJ systems.

Because NHEJ was not able to repair the DSB induced by CRISPR-Cas9, HR system for *R. eutropha* was investigated. Homologous arms of ~ 500 bp were cloned into both pBBR1-Cas9 and pBBR1-Cpf1 to construct the plasmids pBBR1-Cas9-rfpF-rfpR (Additional file 2) and pBBR1-Cpf1-rfpF-rfpR, respectively, which were individually transferred into C5rfp. After induction with 2 mg/mL arabinose for 168 h, the *rfp* genes in all the 23 randomly selected colonies were successfully edited using pBBR1-Cas9-rfpF-rfpR (Table 2, Additional file 1: Figure S1), while no editing occurred in the strain containing pBBR1-Cpf1-rfpF-rfpR (Table 2). Phage-derived recombinases λ-Red [19] and recET [25] were used to enhance the editing efficiency in *E. coli* and *Corynebacterium glutamicum*. In this study, the editing efficiency was 100%, even though none of the heterologous recombinases was expressed, which indicated that *R. eutropha* possesses an efficient endogenous homologous recombination system that supports successful genome editing. Although the CRISPR-Cpf1 system did not work, this is the first report of CRISPR-Cas9-based genome editing in *R. eutropha*. A similar situation was observed in *C. glutamicum* [25, 26], whereby the *cas9* gene from *Streptococcus pyogenes* did not function, while a *cas9* codon-optimized for actinomycetes and *cpf1* from *Francisella tularensis* worked well.

To cure the editing plasmid pBBR1-Cas9-rfpF-rfpR after the editing process, the edited strain was cultured in LB medium without antibiotics for 24 h, after which randomly picked colonies were investigated for resistance against kanamycin. It was found that 98 of 100 colonies lost resistance against kanamycin, which indicated that almost all the cells were cleared of the plasmids (Additional file 1: Figure S2). The high curing efficiency may be due to the instability of the large pBBR1-Cas9-rfpF-rfpR plasmid, which has a low copy number.

**Fig. 3:**
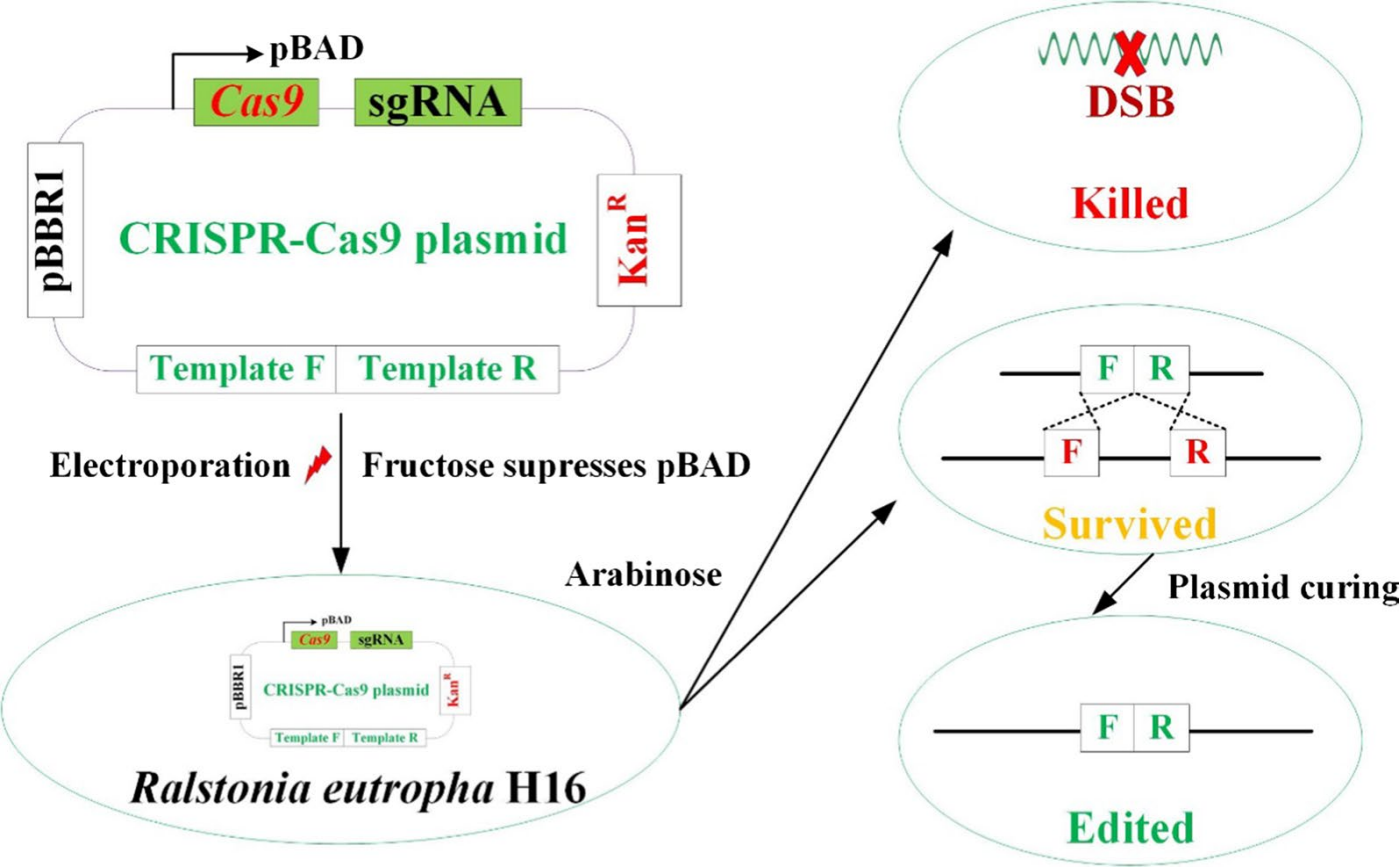
Schematic illustration of the genome editing method for *R. eutropha* using electroporation-based CRISPR-Cas9 technique. The editing plasmid for genome was transferred into *R. eutropha* via electroporation, while 10 mg/mL fructose was added to the regeneration medium in the process of electroporation. Transformants were induced by 2 mg/mL l-arabinose for 96–168 h, then streaked on LB plate with 2 mg/mL l-arabinose and 200 µg/mL kanamycin. Before curing the plasmid, edited strains were identified by PCR and sequencing

In addition, four other *R. eutropha* genes (*H16_A1814*, *H16_A1334*, *H16_A1437* and *H16_B0204*) were selected for editing using the method described above. A schematic illustration is shown in Fig. 3. Induction time was investigated; although the editing efficiency of *H16_A1814* was less than 50% when induction time was 96 h, it increased with the extension of induction time, which reached 78.3% when induction time was extended to 168 h (Table 3). Longer induction time was not investigated, because the activity of the strains decreased sharply. As a result, about 2 weeks were required to edit a single gene, which was faster than the conventional method with an average of 2–3 weeks. All genes in our study were precisely edited as designed, with editing efficiencies ranging from 78.3 to 100% (Table 4, Additional file 1: Figure S3), which was also higher than that of the conventional method with an efficiency lower than 50%.

**Table 3:** Editing efficiencies of *H16_A1814* under different induction times

Induction time (h)	Edited colonies/total colonies	Editing efficiency (%)
96	10/23	43.5
120	13/23	56.5
144	14/23	60.9
168	18/23	78.3

**Table 4:** Editing efficiencies of different loci on the *R. eutropha* genome

Genes	20 bp guide sequence	Deletion size (bp)	Edited colonies/total colonies	Editing efficiency (%)
*rfp*	catgcgtttcaaagttcgta	278	23/23	100
*H16_A1814*	tttcgcgaacctggcaaggt	599	18/23	78.3
*H16_A1334*	ctcggccgccttgctcatgt	330	23/23	100
*H16_A1437*	gcagggacatacggtgtttc	770	18/23	78.3
*H16_B0204*	cagatgccgccgtcgtacag	1031	22/23	95.7

It was found that genes difficult to be disrupted via the conventional methods may be deleted by the CRISPR-Cas9 method. Gene *H16_B0204* failed to be knocked out in our previous work by the conventional pK18mobsacB method, even after several attempts. However, it was successfully knocked out with an efficiency of 95.6% using the CRISPR-Cas9 technique (Table 4, Additional file 1: Figure S3).

Fragment insertion and multigene editing were not performed in this study due to the large size of the editing plasmid, but this shortcoming will be solved by using two compatible broad-host-range plasmids in our future study. Since the plasmid system we used in this research is derived from the broad-host-range plasmid pBBR1MCS2, this method may be applied to other strains or species with minor modifications.

## Conclusions

In summary, an efficient and convenient genome editing method that utilizes the CRISPR-Cas9 system was developed in *R. eutropha* for the first time. Compared to conventional methods, this novel technique significantly increases the efficiency and decreases the time required to manipulate the genome of this important facultative chemolithoautotrophic microbe. The novel technique will facilitate more advanced researches and applications of *R. eutropha* for PHA production and CO_2_ conversion.

## Additional files

**Additional file 1: Figure S1.** *rfp* editing identified by agarose gel electrophoresis and sequencing. **Figure S2.** pBBR1-Cas9-rfpF-rfpR clearance. **Figure S3.** Four genes edited by CRISPR-Cas9. **Table S1.** Putative restriction endonuclease genes in *R. eutropha* H16. **Table S2.** Genes related to putative NHEJ in *R. eutropha.* **Table S3.** List of plasmids used in this study. **Table S4.** List of main primers used in this study.

**Additional file 2.** Profile and sequence of *rfp* editing plasmid pBBR1-Cas9-rfpF-rfpR.

## Abbreviations

PHAs: polyhydroxyalkanoates
RM: restriction modification
HR: homologous recombination
NHEJ: non-homologous end joining
CBB: Calvin–Benson–Bassham
CRISPR: clustered regularly interspaced short palindromic repeats
*rfp*: red fluorescent protein
