# Supplementary material for: Genome editing of Ralstonia eutropha using an electroporation-based CRISPR-Cas9 technique
Source: Biotechnol Biofuels. 2018 Jun 20;11:172. doi: 10.1186/s13068-018-1170-4 (PMC6011247; doi:10.1186/s13068-018-1170-4)
Supplement: Supplementary file 1 — Additional file 1: Figure S1. rfp editing identified by agarose gel electrophoresis and sequencing. Figure S2. pBBR1-Cas9-rfpF-rfpR clearance. Figure S3. Four genes edited by CRISPR-Cas9. Table S1. Putative restriction endonuclease genes in R. eutropha H16. Table S2. Genes related to putative NHEJ in R. eutropha. Table S3. List of plasmids used in this study. Table S4. List of main primers used in this study. [file 13068_2018_1170_MOESM1_ESM.docx]

**Additional file 1**

Genome editing of *Ralstonia eutropha* using an electroporation-based CRISPR-Cas9 technique

Bin Xiong,^1,2,3^ Zhongkang Li,^2,3,4^ Li Liu,^2,3,4^ Dongdong Zhao,^2,3^ Xueli Zhang, ^2,3*^ and Changhao Bi^2,3*^

**Figure S1.** *rfp* editing identified by agarose gel electrophoresis and sequencing.


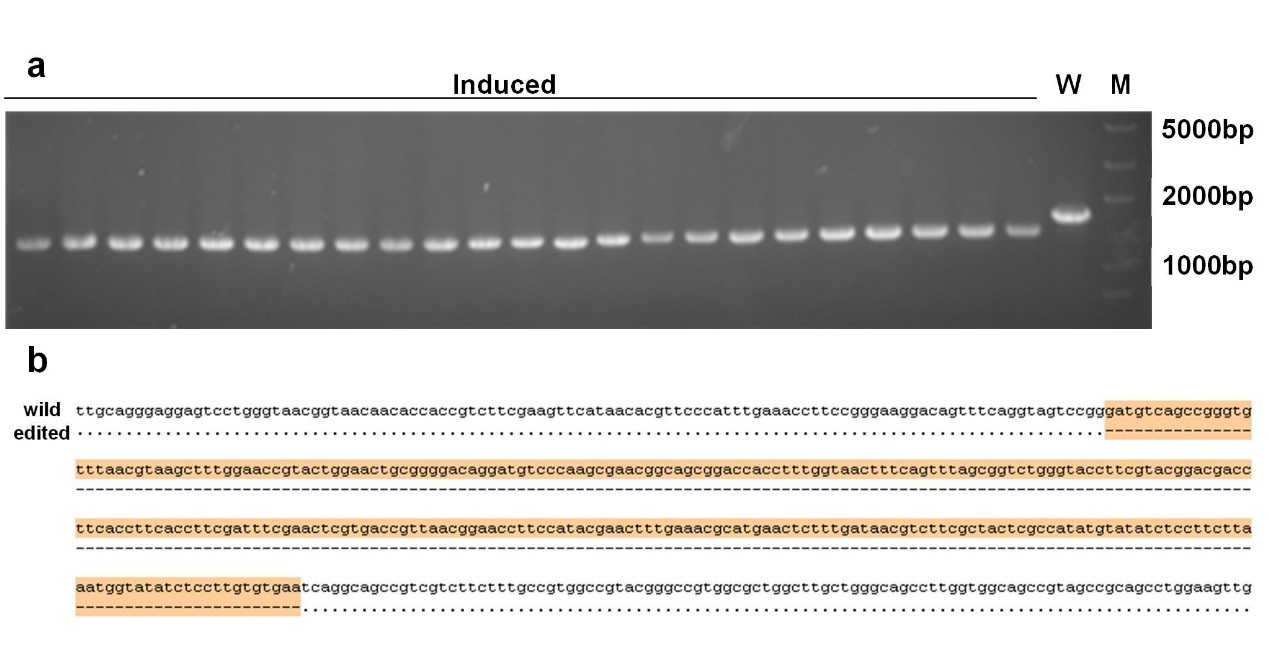


(a) *rfp* editing identified by agarose gel electrophoresis. W: wild type. M: DNA marker (*Trans*2K Plus II, TransGen Biotech, China). (b) *rfp* editing identified by sequencing.

**Figure S2.** pBBR1-Cas9-rfpF-rfpR clearance


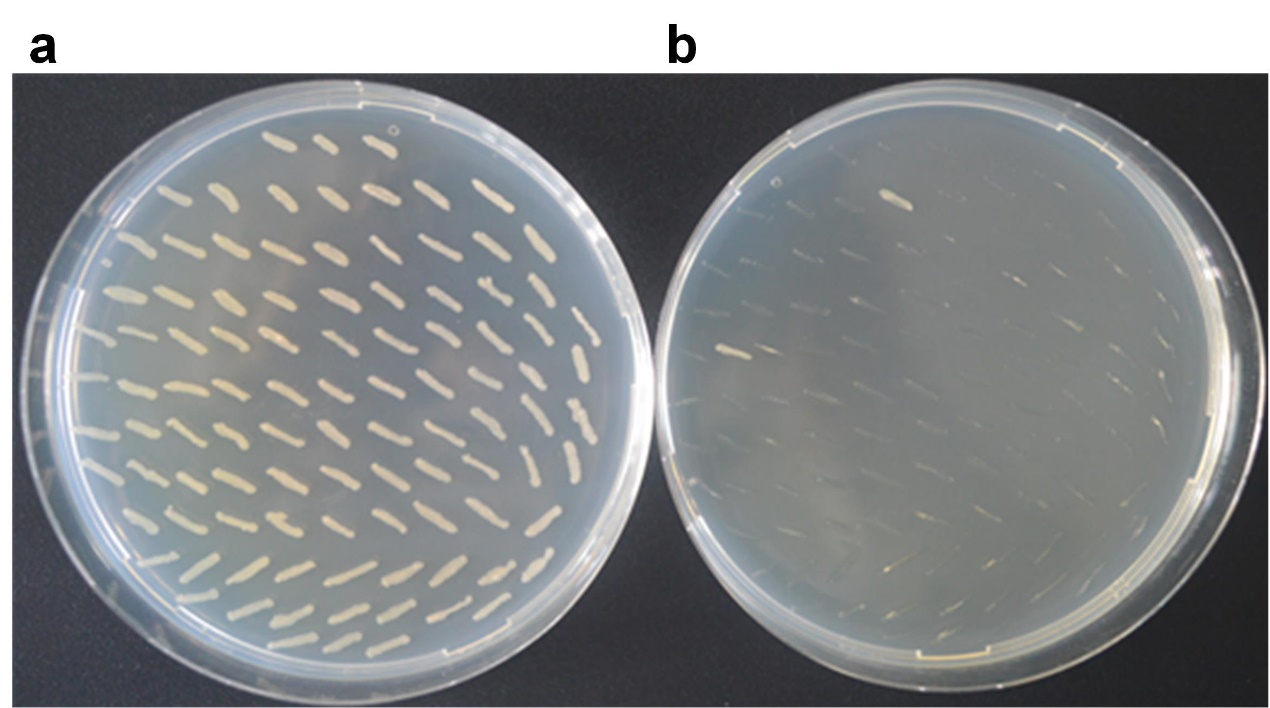


(a) LB plate with no antibiotics, (b) LB plate with 200 µg/mL kanamycin.

**Figure S3.** Four genes edited by CRISPR-Cas9


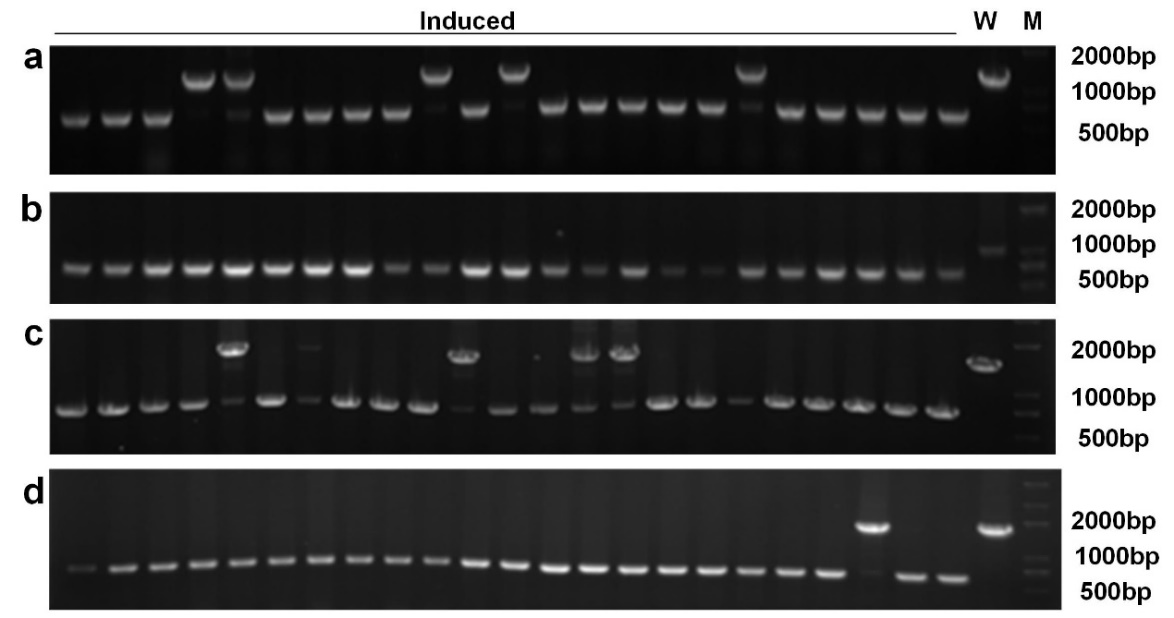


(a) *H16_A1814* editing, (b) *H16_A1334* editing, (c) *H16_A1437* editing, (d) *H16_B0204* editing. W: wild type, M: DNA marker (*Trans*2K Plus II, TransGen Biotech, China).

**Table S1.** Putative restriction endonuclease genes in *R. eutropha* H16

| Putative restriction endonuclease genes in *R. eutropha* H16 | Putative function | Counterpart genes in *E. coli* MG1655 | Amino acids identify |
| --- | --- | --- | --- |
| *H16_A0006* | restriction enzyme | *hsdR* | N^*^ |
| *H16_A0008-9* | 5-methylcytosine restriction enzyme | *mcrBC* | 39% |
| *H16_A0014* | 5-methylcytosine restriction enzyme | *mcrA* | 40% |
| *PHG170* | restriction enzyme | *mmr* | 31% |

*: *H16_A0006* exhibits no similarity to *hsdR* in *E. coli*, while *H16_A0004* exhibits similarity to *hsdM* (a modification subunit of the RM system).

**Table S2.** Genes related to putative NHEJ in *R. eutropha*

| Gene | Putative function | Counterpart genes in *M. tuberculosis* H37Rv | Amino acids identify |
| --- | --- | --- | --- |
| *H16_B2352* | ATP-dependent DNA ligase | *ligD* | 40% |
| *H16_B2355* | non-homologous end-binding protein | *ku70* | 28% |

**Table S3.** List of plasmids used in this study

| plasmid | description | source or reference |
| --- | --- | --- |
| pK18mobsacB  pBBR1MCS2 | plasmid for gene deletion, Km^r^  broad-range host vector used for conjugation, Km^r^ | lab stock  lab stock |
| pBBR1-rfp | derived from pBBR1MCS2, BBa_J23100-*rfp*, Km^r^ | this study |
| pBBR1-pBAD-rfp | derived from pBBR1MCS2, pBAD-*rfp*, Km^r^ | this study |
| pj5_0023 | fragments up-and downstream of *H16_A0006* cloned into pK18mobsacB | this study |
| pj5_0025 | fragments up-and downstream of *H16_A0008-9* cloned into pK18mobsacB | this study |
| pj5_0026 | fragments up-and downstream of *H16_A0014* cloned into pK18mobsacB | this study |
| pj5_0027 | fragments up-and downstream of *PHG170* cloned into pK18mobsacB | this study |
| pj5_0021 | two fragments downstream of *phaP1* between which *rfp* with BBa_J23100 and an RBS was inserted cloned into pK18mobsacB | this study |
| pBBR1-Cas9 | derived from pBBR1-pBAD-rfp, harboring CRISPR-Cas9 system | this study |
| pBBR1-Cpf1 | derived from pBBR1-pBAD-rfp, harboring CRISPR-Cpf1 system | this study |
| pBBR1-Cas9-ligD-ku | derived from pBBR1-Cas9, harboring CRISPR-Cas9 and *ligD*-*ku70* | this study |
| pBBR1-Cpf1-ligD-ku | derived from pBBR1-Cpf1, harboring CRISPR-Cpf1 and *ligD*-*ku70* | this study |
| pBBR1-Cas9-rfpF-rfpR | derived from pBBR1-Cas9, harboring CRISPR-Cas9 and template, for *rfp* editing | this study |
| pBBR1-Cpf1-rfpF-rfpR | derived from pBBR1-Cpf1, harboring CRISPR-Cpf1 and template, for *rfp* editing | this study |
| pBBR1-Cas9-A1437F-A1437R | derived from pBBR1-Cas9, harboring CRISPR-Cas9 and template, for *H16_A1437* editing | this study |
| pBBR1-Cas9-B0204F-B0204R | derived from pBBR1-Cas9, harboring CRISPR-Cas9 and template, for *H16_B0204* editing | this study |
| pBBR1-Cas9-A1334F-A1334R | derived from pBBR1-Cas9, harboring CRISPR-Cas9 and template, for *H16_A1334* editing | this study |
| pBBR1-Cas9-A1814F-A1814R | derived from pBBR1-Cas9, harboring CRISPR-Cas9 and template, for *H16_A1814* editing | this study |

**Table S4.** List of main primers used in this study

| primer | sequence |
| --- | --- |
| pj5_0023 construction, *H16_A0006* deletion | |
| j5_00059_pK18_f | CACACCAGGTCTCAAACTCACATTAATTGCGTTGCG |
| j5_00060_pK18_r | CACACCAGGTCTCAGCTGCTAAAGGAAGCGGAACACG |
| j5_00061_hsdR_f | CACACCAGGTCTCACAGCAAGCACGTTTCGAGTTAGCCTTGC |
| j5_00062_hsdR_r | CACACCAGGTCTCACTTCAGGCGCTCCCTGCTTGTTTGG |
| j5_00063_hsdR_f | CACACCAGGTCTCAGAAGCTGATTGATCAGGCTCGCGCC |
| j5_00064_hsdR_r | CACACCAGGTCTCAAGTTCGCGACTGGTAGTCAGCTACACGC |
| C1_4450F | GCAGGGTGGCGCGGGAATTAG |
| C1_4450R | CGGCAGCAGGATTGCCTTCCAG |
| pj5_0025 construction, *H16_A0008-9* deletion | |
| j5_00040_pK18_f | CACACCAGGTCTCACTACTCACATTAATTGCGTTGCG |
| j5_00021_pK18_r | CACACCAGGTCTCACGTGCTAAAGGAAGCGGAACACG |
| j5_00066_mcrBC_f | CACACCAGGTCTCACACGCCAGGTTCCGCGCAAGG |
| j5_00070_mcrBC_r | CACACCAGGTCTCAGGCGTTCTTTAGATTCCTTCAACATCGATTCCATGC |
| j5_00071_mcrBC_f | CACACCAGGTCTCACGCCAAGCACCTGGCGAAACAGCTG |
| j5_00072_mcrBC_r | CACACCAGGTCTCAGTAGCGGACTCATCCGATCAGACTTGG |
| C2_4496F | GCATTCGTGCTGGAGAAG |
| C2_4496R | TACGAGTGTGCGTCTAGG |
| pj5_0026 construction, *H16_A0014* deletion | |
| j5_00073_pK18_f | CACACCAGGTCTCATCACTCACATTAATTGCGTTGCG |
| j5_00044_pK18_r | CACACCAGGTCTCATTTGCTAAAGGAAGCGGAACACG |
| j5_00074_mcrA_f | CACACCAGGTCTCACAAATGTCTGCGGAACCAAAATTACG |
| j5_00075_mcrA_r | CACACCAGGTCTCACGACGCAAGAGCTGGAGATTACCCG |
| j5_00076_mcrA_f | CACACCAGGTCTCAGTCGGCTTTCTTTCGACATCGTAG |
| j5_00077_mcrA_r | CACACCAGGTCTCAGTGACTGCATCATGACCTCCGG |
| C3_2310F | CCGTGCGTTCGCGGTAATTGGG |
| C3_2310R | TTGGGCTAGGGTGGGCTCTTGG |
| pj5_0027 construction, *PHG170* deletion | |
| j5_00042_pK18_f | CACACCAGGTCTCAGCACTCACATTAATTGCGTTGCG |
| j5_00044_pK18_r | CACACCAGGTCTCATTTGCTAAAGGAAGCGGAACACG |
| j5_00078_PHG170_f | CACACCAGGTCTCACAAACCCCAAGGCCGCCAGACC |
| j5_00079_PHG170_r | CACACCAGGTCTCAGATTCTCTACCTGTTGTTGTTCTTCATCTCATTGGCG |
| j5_00080_PHG170_f | CACACCAGGTCTCAAATCTAGGAACCGGCTCGATCAATCATC |
| j5_00081_PHG170_r | CACACCAGGTCTCAGTGCGATAGGCAGATGGGTTCCGGC |
| C4_2157F | GCGTAAGCGGAGAACGTAATG |
| C4_2157R | GTCGAGGGCGATAAAGAAAGC |
| pj5_0021 construction, *rfp* insertion | |
| j5_00027_pj5_00006_f | TGTATATCTCCTTCTTAAATGGTATATCTCCTTGTGTGAATCAGGCAGCC |
| j5_00019_pj5_00006_r | GTGCTTAAGGATAACTGCCTGCGTTGAAGATGGACCG |
| j5_00006_rfp_f | CAACGCAGGCAGTTATCCTTAAGCACCGGTGGAGTG |
| j5_00028_rfp_r | GATTCACACAAGGAGATATACCATTTAAGAAGGAGATATACATATGGCGAG |
| C5rfp_1144F | CCGCTGCACTTCACCGCATTCC |
| C5rfp_1144R | CGAAACGCTGTTCGGCCTGACC |
| pBBR1-Cas9 construction | |
| j5_00017_pBAD_f | CACACCAGGTCTCAGGGGTGCTTAAGGATCCAAACTCG |
| j5_00002_pBAD_r | CACACCAGGTCTCATATGTATATCTCCTTCTTAAAAGATCTTTTGAATTCC |
| j5_00013_pj5_0033_f | CACACCAGGTCTCACATATGGATAAGAAATACTCAATAGGCTTAGATATCGGC |
| j5_00025_pj5_0033_r | CACACCAGGTCTCACTTTGAAACGCATGGCTAAGATCTGACTCCATAACAGAGTACTCGCC |
| j5_00026_pj5_0033_f | CACACCAGGTCTCAAAAGTTCGTAGTTTTAGAGCTAGAAATAGCAAGTTAAAATAAGGC |
| j5_00019_pj5_0033_r | CACACCAGGTCTCACCCCTAGGTATAAACGCAGAAAGGCCC |
| pBBR1-Cpf1 construction | |
| j5_00010_pBAD_f | CACACCAGGTCTCAGCACACCGGGCGTTTTTTCTTTGTGAGTCCACCAGGCATCAAATAAAACGAAAGGC |
| j5_00002_pBAD_r | CACACCAGGTCTCATATGTATATCTCCTTCTTAAAAGATCTTTTGAATTCC |
| j5_00008_Exported_f | CACACCAGGTCTCACATATGTCAATTTATCAAGAATTTGTTAATAAATATAGT |
| j5_00011_Exported_r | CACACCAGGTCTCAGTGCAAGACCGAGCGTTCTGAACAAATTTAAAGTTCTTAGACTTTTAACAGTGGCC |
| pBBR1-Cas9-ligD-ku and pBBR1-Cpf1-ligD-ku construction | |
| j5_00046_pj5_0044_f | CACACCAGGTCTCAGATTCCATTCAGGTCGAGGTGGCCC |
| j5_00047_pj5_0044_r | CACACCAGGTCTCATTATACCTAGGACTGAGCTAGCTGTCAAGAGCTCTCGAACCCCAGAGTCCC |
| j5_00048_(ku-ligD)_f | CACACCAGGTCTCAATAATGCTAGCTTTAAGAAGGAGATATACATATGGGTTCGGCGTCGGAGC |
| j5_00043_(ku-ligD)_r | CACACCAGGTCTCACTCCTTCTTAAATCATTCGCGCACCACCTCACTGG |
| j5_00044_(ku-ligD)_f | CACACCAGGTCTCAGGAGATATACATATGCGAGCCATTTGGACGGG |
| j5_00049_(ku-ligD)_r | CACACCAGGTCTCAAATCACGGAGGCGTTGGGAC |
| pBBR1-Cas9-rfpF-rfpR and pBBR1-Cpf1-rfpF-rfpR construction, *rfp* editing | |
| j5_00030_pj5_0044_f | CACACCAGGTCTCAGTGAGCTCTCGAACCCCAGAGTCCC |
| j5_00025_pj5_0044_r | CACACCAGGTCTCACGTTCCATTCAGGTCGAGGTGGCCC |
| j5_00026_RFP_f | CACACCAGGTCTCAAACGCGGTACCGAGGAGACATCG |
| j5_00031_RFP_r | CACACCAGGTCTCAGACCGGACTACCTGAAACTGTCCTTCCCG |
| j5_00032_rfp_f | CACACCAGGTCTCAGGTCAGGCAGCCGTCGTCTTCTTTGC |
| j5_00033_rfp_r | CACACCAGGTCTCATCACCAGCAATGATCCTCACCCCGG |
| *rfp*_1673F | TGTTCACCGGGACCAACCTG |
| *rfp*_1673R | GAAATGCGCCTTGACCCACC |
| pBBR1-Cas9-A1437F-A1437R construction, *H16_A1437* editing | |
| j5_00001_pj5_0046_f | CACACCAGGTCTCAGAGAGCTCTCGAACCCCAGAGTCCC |
| j5_00020_pj5_0046_r | CACACCAGGTCTCACGTATGTCCCTGCGCTAAGATCTGACTCCATAACAGAGTACTCGCC |
| j5_00021_(46-44)_f | CACACCAGGTCTCATACGGTGTTTCGTTTTAGAGCTAGAAATAGCAAGTTAAAATAAGGC |
| j5_00016_(46-44)_r | CACACCAGGTCTCATTTTCCATTCAGGTCGAGGTGGCC |
| j5_00017_phaC1_f | CACACCAGGTCTCAAAAAAGGCGCGGCAGCTTCC |
| j5_00018_phaC1_r | CACACCAGGTCTCACCTGCGCCTCGGGATTGGTGG |
| j5_00019_phaC1_f | CACACCAGGTCTCACAGGAACGGCGACGCCACCAACC |
| j5_00008_phaC1_r | CACACCAGGTCTCATCTCATGCCTTGGCTTTGACGTATCGCC |
| A1437_1981F | AGCCGGGTCCATTCGGATAG |
| A1437_916R | TGAGCTCGTTCTGCAGGTAGG |
| pBBR1-Cas9-A1814F-A1814R construction, *H16_A1814* editing | |
| j5_00040_pj5_0046_f | CACACCAGGTCTCACGAGCTCTCGAACCCCAGAGTCCC |
| j5_00041_pj5_0046_r | CACACCAGGTCTCACAGGTTCGCGAAAGCTAAGATCTGACTCCATAACAGAGTACTCGCC |
| j5_00042_(46-44)_f | CACACCAGGTCTCACCTGGCAAGGTGTTTTAGAGCTAGAAATAGCAAGTTAAAATAAGGC |
| j5_00043_(46-44)_r | CACACCAGGTCTCAGATTCCATTCAGGTCGAGGTGGCC |
| j5_00044_(A1814F)_f | CACACCAGGTCTCAAATCCCATGGCAGCTTGCGG |
| j5_00045_(A1814F)_r | CACACCAGGTCTCAGACGCACCGGATCTCGCCCG |
| j5_00046_(A1814R)_f | CACACCAGGTCTCACGTCAAGCATGTTGATCTCCTGTTAGCGTGGG |
| j5_00047_(A1814R)_r | CACACCAGGTCTCACTCGTGGCCAACGCCGCCACC |
| A1814 _1217F | AGGACATCGCGCACGACG |
| A1814 _1217R | GCGGACGCATCGAGACCAAC |
| pBBR1-Cas9-A1334F-A1334R construction, *H16_A1334* editing | |
| j5_00001_pj5_0046_f | CACACCAGGTCTCAGTGAGCTCTCGAACCCCAGAGTCCC |
| j5_00048_pj5_0046_r | CACACCAGGTCTCACAAGGCGGCCGAGGCTAAGATCTGACTCCATAACAGAGTACTCGCC |
| j5_00049_(46-44)_f | CACACCAGGTCTCACTTGCTCATGTGTTTTAGAGCTAGAAATAGCAAGTTAAAATAAGGC |
| j5_00050_(46-44)_r | CACACCAGGTCTCATTTCCATTCAGGTCGAGGTGGCC |
| j5_00051_(A1334F)_f | CACACCAGGTCTCAGAAAGCGCGCGCAGGAAACTGC |
| j5_00015_(A1334F)_r | CACACCAGGTCTCAACATCAACCTGACCTCGGCCTTCC |
| j5_00012_(A1334R)_f | CACACCAGGTCTCAATGTAAGCCTCCGTCGAGAGAAAGG |
| j5_00008_(A1334R)_r | CACACCAGGTCTCATCACGTTGAGCCGCGCAATCAGC |
| A1334_961F | GGGCTCTTGTAGACGTCGATG |
| A1334_961R | CCCTGTGGCAACCTTTCTGTG |
| pBBR1-Cas9-B0204F-B0204R construction, *H16_B0204* editing | |
| j5_00079_pj5_0046_f | CACACCAGGTCTCAGGCGAGCTCTCGAACCCCAGAGTCCC |
| j5_00064_pj5_0046_r | CACACCAGGTCTCAACGGCGGCATCTGGCTAAGATCTGACTCCATAACAGAGTACTCGCC |
| j5_00065_(46-44)_f | CACACCAGGTCTCACCGTCGTACAGGTTTTAGAGCTAGAAATAGCAAGTTAAAATAAGGC |
| j5_00071_(46-44)_r | CACACCAGGTCTCAGTTCCATTCAGGTCGAGGTGGCC |
| j5_00072_(B0204F)_f | CACACCAGGTCTCAGAACGTGTTGTGCGGGAATACCGCGTAG |
| j5_00073_(B0204F)_r | CACACCAGGTCTCACTCGCGAGGCCGCGCAACTG |
| j5_00080_(B0204R)_f | CACACCAGGTCTCACGAGAGCCTTGCCGCCACCCATGAAGAAGG |
| j5_00081_(B0204R)_r | CACACCAGGTCTCACGCCGGCTGTGCCCCGGCC |
| B0204_1661F | GCCGGCAGAGACATCACG |
| B0204_1661R | GCGCACCAACGCCTTCTTC |
