# Supplementary material for: Genome editing of Ralstonia eutropha using an electroporation-based CRISPR-Cas9 technique
Source: Biotechnol Biofuels. 2018 Jun 20;11:172. doi: 10.1186/s13068-018-1170-4 (PMC6011247; doi:10.1186/s13068-018-1170-4)
Supplement: Supplementary file 2 — Additional file 2. Profile and sequence of rfp editing plasmid pBBR1-Cas9-rfpF-rfpR. [file 13068_2018_1170_MOESM2_ESM.docx]

**Profile and sequence of plasmid pBBR1-Cas9-rfpF-rfpR**

**Profile of** **plasmid pBBR1-Cas9-rfpF-rfpR**


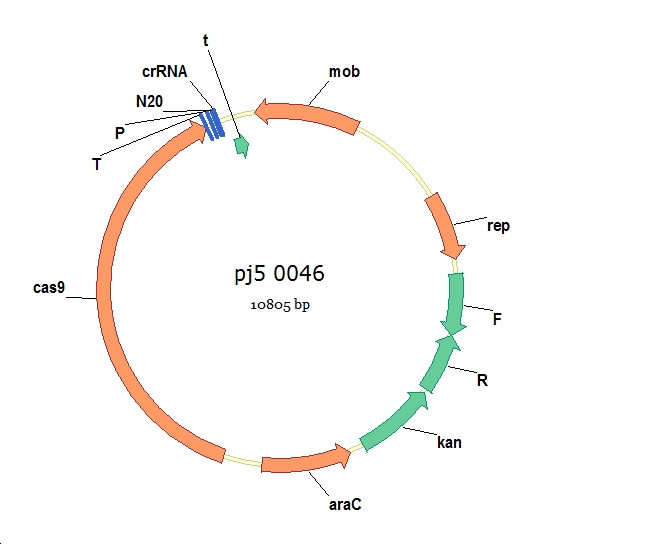


**Sequence of plasmid pBBR1-Cas9-rfpF-rfpR**

gctcctgcggcggcctgtagggcaggctcatacccctgccgaaccgcttttgtcagccggtcggccacggcttccggcgtctcaacgcgctttgagattcccagcttttcggccaatccctgcggtgcataggcgcgtggctcgaccgcttgcgggctgatggtgacgtggcccactggtggccgctccagggcctcgtagaacgcctgaatgcgcgtgtgacgtgccttgctgccctcgatgccccgttgcagccctagatcggccacagcggccgcaaacgtggtctggtcgcgggtcatctgcgctttgttgccgatgaactccttggccgacagcctgccgtcctgcgtcagcggcaccacgaacgcggtcatgtgcgggctggtttcgtcacggtggatgctggccgtcacgatgcgatccgccccgtacttgtccgccagccacttgtgcgccttctcgaagaacgccgcctgctgttcttggctggccgacttccaccattccgggctggccgtcatgacgtactcgaccgccaacacagcgtccttgcgccgcttctctggcagcaactcgcgcagtcggcccatcgcttcatcggtgctgctggccgcccagtgctcgttctctggcgtcctgctggcgtcagcgttgggcgtctcgcgctcgcggtaggcgtgcttgagactggccgccacgttgcccattttcgccagcttcttgcatcgcatgatcgcgtatgccgccatgcctgcccctcccttttggtgtccaaccggctcgacgggggcagcgcaaggcggtgcctccggcgggccactcaatgcttgagtatactcactagactttgcttcgcaaagtcgtgaccgcctacggcggctgcggcgccctacgggcttgctctccgggcttcgccctgcgcggtcgctgcgctcccttgccagcccgtggatatgtggacgatggccgcgagcggccaccggctggctcgcttcgctcggcccgtggacaaccctgctggacaagctgatggacaggctgcgcctgcccacgagcttgaccacagggattgcccaccggctacccagccttcgaccacatacccaccggctccaactgcgcggcctgcggccttgccccatcaatttttttaattttctctggggaaaagcctccggcctgcggcctgcgcgcttcgcttgccggttggacaccaagtggaaggcgggtcaaggctcgcgcagcgaccgcgcagcggcttggccttgacgcgcctggaacgacccaagcctatgcgagtgggggcagtcgaaggcgaagcccgcccgcctgccccccgagcctcacggcggcgagtgcgggggttccaagggggcagcgccaccttgggcaaggccgaaggccgcgcagtcgatcaacaagccccggaggggccactttttgccggagggggagccgcgccgaaggcgtgggggaaccccgcaggggtgcccttctttgggcaccaaagaactagatatagggcgaaatgcgaaagacttaaaaatcaacaacttaaaaaaggggggtacgcaacagctcattgcggcaccccccgcaatagctcattgcgtaggttaaagaaaatctgtaattgactgccacttttacgcaacgcataattgttgtcgcgctgccgaaaagttgcagctgattgcgcatggtgccgcaaccgtgcggcaccctaccgcatggagataagcatggccacgcagtccagagaaatcggcattcaagccaagaacaagcccggtcactgggtgcaaacggaacgcaaagcgcatgaggcgtgggccgggcttattgcgaggaaacccacggcggcaatgctgctgcatcacctcgtggcgcagatgggccaccagaacgccgtggtggtcagccagaagacactttccaagctcatcggacgttctttgcggacggtccaatacgcagtcaaggacttggtggccgagcgctggatctccgtcgtgaagctcaacggccccggcaccgtgtcggcctacgtggtcaatgaccgcgtggcgtggggccagccccgcgaccagttgcgcctgtcggtgttcagtgccgccgtggtggttgatcacgacgaccaggacgaatcgctgttggggcatggcgacctgcgccgcatcccgaccctgtatccgggcgagcagcaactaccgaccggccccggcgaggagccgcccagccagcccggcattccgggcatggaaccagacctgccagccttgaccgaaacggaggaatgggaacggcgcgggcagcagcgcctgccgatgcccgatgagccgtgttttctggacgatggcgagccgttggagccgccgacacgggtcacgctgccgcgccggtagcacttgggttgcgcagcaacccgtaagtgcgctgttccagactatcggctgtagccgcctcgccgccctataccttgtctgcctccccgcgttgcgtcgcggtgcatggagccgggccacctcgacctgaatggaacgcggtaccgaggagacatcggcggacaaggcagcaccgatgcaactcagtcagggcactaccttcatcgttggcaaaacacaccgcaaacgccaggcgtcgatatgctttgccaacggaccggccgcagccggtccatcttcaacgcaggcagttatccttaagcaccggtggagtgacgaccttcagcacgttcgtactgttcaacgatggtgtagtcttcgttgtgggaggtgatgtccagtttgatgtcggttttgtaagcacccggcagctgaaccggttttttagccatgtaggtggttttaacttcagcgtcgtagtgaccaccgtctttcagtttcagacgcattttgatttcacctttcagagcaccgtcttccgggtacatacgttcggtggaagcttcccaacccatggtttttttctgcataaccggaccgtcggacgggaagttggtaccacgcagtttaactttgtagatgaactcaccgtcttgcagggaggagtcctgggtaacggtaacaacaccaccgtcttcgaagttcataacacgttcccatttgaaaccttccgggaaggacagtttcaggtagtccggtcaggcagccgtcgtcttctttgccgtggccgtacgggccgtggcgctggcttgctgggcagccttggtggcagccgtagccgcagcctggaagttggtttcagcgatttcgaccgcttgcttggtcgccttctgcaccgactcgtaggcgttgttggcagcggagatcgccgacttcacgatggccacggtcgattccgaaccggccggggcgttcttggcgaggttctcgaccagcgcttgcacgttcttcgagccttcggccagttgagcctcggctaccttggtgaactcgctctgggtttccgaagcgatttcatacaggtggcgggtgtaggccagggtcttttcggcaaccggctgcacggctgcggcctggatggccagcagttcctgtgcgtccttggccgacagcgccttcttggcgttgtcaacgccttctgcgaacgaagtcttgacgacctgcaggttcagctcgacgagcttttcgacgccttcaaacgccttggtggtcaggccgaacagcgtttcgaggttggccttttgcgctgctgcaacttgttccggggtgaggatcattgctggtgagctctcgaaccccagagtcccgctcagaagaactcgtcaagaaggcgatagaaggcgatgcgctgcgaatcgggagcggcgataccgtaaagcacgaggaagcggtcagcccattcgccgccaagctcttcagcaatatcacgggtagccaacgctatgtcctgatagcggtccgccacacccagccggccacagtcgatgaatccagaaaagcggccattttccaccatgatattcggcaagcaggcatcgccatgggtcacgacgagatcctcgccgtcgggcatgcgcgccttgagcctggcgaacagttcggctggcgcgagcccctgatgctcttcgtccagatcatcctgatcgacaagaccggcttccatccgagtacgtgctcgctcgatgcgatgtttcgcttggtggtcgaatgggcaggtagccggatcaagcgtatgcagccgccgcattgcatcagccatgatggatactttctcggcaggagcaaggtgagatgacaggagatcctgccccggcacttcgcccaatagcagccagtcccttcccgcttcagtgacaacgtcgagcacagctgcgcaaggaacgcccgtcgtggccagccacgatagccgcgctgcctcgtcctgcagttcattcagggcaccggacaggtcggtcttgacaaaaagaaccgggcgcccctgcgctgacagccggaacacggcggcatcagagcagccgattgtctgttgtgcccagtcatagccgaatagcctctccacccaagcggccggagaacctgcgtgcaatccatcttgttcaatcatgcgaaacgatcctcatcctgtctcttgatcagatcatgatcccctgcgccatcagatccttggcggcaagaaagccatccagtttactttgcagggcttcccaaccttaccagagggcgccccagctggcaattccgacgtcttatgacaacttgacggctacatcattcactttttcttcacaaccggcacggaactcgctcgggctggccccggtgcattttttaaatacccgcgagaaatagagttgatcgtcaaaaccaacattgcgaccgacggtggcgataggcatccgggtggtgctcaaaagcagcttcgcctggctgatacgttggtcctcgcgccagcttaagacgctaatccctaactgctggcggaaaagatgtgacagacgcgacggcgacaagcaaacatgctgtgcgacgctggcgatatcaaaattgctgtctgccaggtgatcgctgatgtactgacaagcctcgcgtacccgattatccatcggtggatggagcgactcgttaatcgcttccatgcgccgcagtaacaattgctcaagcagatttatcgccagcagctccgaatagcgcccttccccttgcccggcgttaatgatttgcccaaacaggtcgctgaaatgcggctggtgcgcttcatccgggcgaaagaaccccgtattggcaaatattgacggccagttaagccattcatgccagtaggcgcgcggacgaaagtaaacccactggtgataccattcgcgagcctccggatgacgaccgtagtgatgaatctctcctggcgggaacagcaaaatatcacccggtcggcaaacaaattctcgtccctgatttttcaccaccccctgaccgcgaatggtgagattgagaatataacctttcattcccagcggtcggtcgataaaaaaatcgagataaccgttggcctcaatcggcgttaaacccgccaccagatgggcattaaacgagtatcccggcagcaggggatcattttgcgcttcagccatacttttcatactcccgccattcagagaagaaaccaattgtccatattgcatcagacattgccgtcactgcgtcttttactggctcttctcgctaaccaaaccggtaaccccgcttattaaaagcattctgtaacaaagcgggaccaaagccatgacaaaaacgcgtaacaaaagtgtctataatcacggcagaaaagtccacattgattatttgcacggcgtcacactttgctatgccatagcatttttatccataagattagcggattctacctgacgctttttatcgcaactctctactgtttctccatagggagtccacaacggtttccctctagaaataattttggaattcaaaagatcttttaagaaggagatatacatatggataagaaatactcaataggcttagatatcggcacaaatagcgtcggatgggcggtgatcactgatgaatataaggttccgtctaaaaagttcaaggttctgggaaatacagaccgccacagtatcaaaaaaaatcttataggggctcttttatttgacagtggagagacagcggaagcgactcgtctcaaacggacagctcgtagaaggtatacacgtcggaagaatcgtatttgttatctacaggagattttttcaaatgagatggcgaaagtagatgatagtttctttcatcgacttgaagagtcttttttggtggaagaagacaagaagcatgaacgtcatcctatttttggaaatatagtagatgaagttgcttatcatgagaaatatccaactatctatcatctgcgaaaaaaattggtagattctactgataaagcggatttgcgcttaatctatttggccttagcgcatatgattaagtttcgtggtcattttttgattgagggagatttaaatcctgataatagtgatgtggacaaactatttatccagttggtacaaacctacaatcaattatttgaagaaaaccctattaacgcaagtggagtagatgctaaagcgattctttctgcacgattgagtaaatcaagacgattagaaaatctcattgctcagctccccggtgagaagaaaaatggcttatttgggaatctcattgctttgtcattgggtttgacccctaattttaaatcaaattttgatttggcagaagatgctaaattacagctttcaaaagatacttacgatgatgatttagataatttattggcgcaaattggagatcaatatgctgatttgtttttggcagctaagaatttatcagatgctattttactttcagatatcctaagagtaaatactgaaataactaaggctcccctatcagcttcaatgattaaacgctacgatgaacatcatcaagacttgactcttttaaaagctttagttcgacaacaacttccagaaaagtataaagaaatcttttttgatcaatcaaaaaacggatatgcaggttatattgatgggggagctagccaagaagaattttataaatttatcaaaccaattttagaaaaaatggatggtactgaggaattattggtgaaactaaatcgtgaagatttgctgcgcaagcaacggacctttgacaacggctctattccccatcaaattcacttgggtgagctgcatgctattttgagaagacaagaagacttttatccatttttaaaagacaatcgtgagaagattgaaaaaatcttgacttttcgaattccttattatgttggtccattggcgcgtggcaatagtcgttttgcatggatgactcggaagtctgaagaaacaattaccccatggaattttgaagaagttgtcgataaaggtgcttcagctcaatcatttattgaacgcatgacaaactttgataaaaatcttccaaatgaaaaagtactaccaaaacatagtttgctttatgagtattttacggtttataacgaattgacaaaggtcaaatatgttactgaaggaatgcgaaaaccagcatttctttcaggtgaacagaagaaagccattgttgatttactcttcaaaacaaatcgaaaagtaaccgttaagcaattaaaagaagattatttcaaaaaaatagaatgttttgatagtgttgaaatttcaggagttgaagatagatttaatgcttcattaggtacctaccatgatttgctaaaaattattaaagataaagattttttggataatgaagaaaatgaagatatcttagaggatattgttttaacattgaccttatttgaagatagggagatgattgaggaaagacttaaaacatatgctcacctctttgatgataaggtgatgaaacagcttaaacgtcgccgttatactggttggggacgtttgtctcgaaaattgattaatggtattagggataagcaatctggcaaaacaatattagattttttgaaatcagatggttttgccaatcgcaattttatgcagctgatccatgatgatagtttgacatttaaagaagacattcaaaaagcacaagtgtctggacaaggcgatagtttacatgaacatattgcaaatttagctggtagccctgctattaaaaaaggtattttacagactgtaaaagttgttgatgaattggtcaaagtaatggggcggcataagccagaaaatatcgttattgaaatggcacgtgaaaatcagacaactcaaaagggccagaaaaattcgcgagagcgtatgaaacgaatcgaagaaggtatcaaagaattaggaagtcagattcttaaagagcatcctgttgaaaatactcaattgcaaaatgaaaagctctatctctattatctccaaaatggaagagacatgtatgtggaccaagaattagatattaatcgtttaagtgattatgatgtcgatcacattgttccacaaagtttccttaaagacgattcaatagacaataaggtcttaacgcgttctgataaaaatcgtggtaaatcggataacgttccaagtgaagaagtagtcaaaaagatgaaaaactattggagacaacttctaaacgccaagttaatcactcaacgtaagtttgataatttaacgaaagctgaacgtggaggtttgagtgaacttgataaagctggttttatcaaacgccaattggttgaaactcgccaaatcactaagcatgtggcacaaattttggatagtcgcatgaatactaaatacgatgaaaatgataaacttattcgagaggttaaagtgattaccttaaaatctaaattagtttctgacttccgaaaagatttccaattctataaagtacgtgagattaacaattaccatcatgcccatgatgcgtatctaaatgccgtcgttggaactgctttgattaagaaatatccaaaacttgaatcggagtttgtctatggtgattataaagtttatgatgttcgtaaaatgattgctaagtctgagcaagaaataggcaaagcaaccgcaaaatatttcttttactctaatatcatgaacttcttcaaaacagaaattacacttgcaaatggagagattcgcaaacgccctctaatcgaaactaatggggaaactggagaaattgtctgggataaagggcgagattttgccacagtgcgcaaagtattgtccatgccccaagtcaatattgtcaagaaaacagaagtacagacaggcggattctccaaggagtcaattttaccaaaaagaaattcggacaagcttattgctcgtaaaaaagactgggatccaaaaaaatatggtggttttgatagtccaacggtagcttattcagtcctagtggttgctaaggtggaaaaagggaaatcgaagaagttaaaatccgttaaagagttactagggatcacaattatggaaagaagttcctttgaaaaaaatccgattgactttttagaagctaaaggatataaggaagttaaaaaagacttaatcattaaactacctaaatatagtctttttgagttagaaaacggtcgtaaacggatgctggctagtgccggagaattacaaaaaggaaatgagctggctctgccaagcaaatatgtgaattttttatatttagctagtcattatgaaaagttgaagggtagtccagaagataacgaacaaaaacaattgtttgtggagcagcataagcattatttagatgagattattgagcaaatcagtgaattttctaagcgtgttattttagcagatgccaatttagataaagttcttagtgcatataacaaacatagagacaaaccaatacgtgaacaagcagaaaatattattcatttatttacgttgacgaatcttggagctcccgctgcttttaaatattttgatacaacaattgatcgtaaacgatatacgtctacaaaagaagttttagatgccactcttatccatcaatccatcactggtctttatgaaacacgcattgatttgagtcagctaggaggtgactgagcatcaaataaaacgaaaggctcagtcgaaagactgggcctttcgttttatctgtctaggtttatacataggcgagtactctgttatggagtcagatcttagccatgcgtttcaaagttcgtagttttagagctagaaatagcaagttaaaataaggctagtccgttatcaacttgaaaaagtggcaccgagtcggtgcttagcatccaaactcgagtaaggatctccaggcatcaaataaaacgaaaggctcagtcgaaagactgggcctttcgttttatctgttgtttgtcggtgaacgctctctactagagtcacactggctcaccttcgggtgggcctttctgcgtttatacctaggggtgcttaaggatccaaactcgagtaaggatctccaggcatcaaataaaacgaaaggctcagtcgaaagactgggcctttcgttttatctgttgtttgtcggtgaacgctctctactagagtcacactggctcaccttcgggtgggcctttctgcgtttatacctagggcgttcggctgcggctggcgctgggcctgtttctggcgctggacttcccgctgttccgtcagcagcttttcgcccacggccttgatgatcgcggcggccttggcctgcatatcccgattcaacggccccagggcgtccagaacgggcttcaggcgctcccgaagatctcgggccgtctcttgggcttgatcggccttcttgcgcatctcacgc
